# Bacterial colonization of Montgomery salivary bypass tubes after hypopharyngeal reconstruction in head and neck cancer patients

**DOI:** 10.1007/s00405-019-05768-z

**Published:** 2019-12-17

**Authors:** Stefan Grasl, Stefan Janik, Matthaeus Christoph Grasl, Bernhard Parschalk, Boban M. Erovic, Georg Haymerle

**Affiliations:** 1 Department of Otorhinolaryngology, Head and Neck Surgery, Medical University of Vienna, Waehringer Guertel 18-20, 1090 Vienna, Austria; 2 Institute of Head and Neck Diseases, Evangelical Hospital Vienna, Vienna, Austria

**Keywords:** Bacterial colonization, Montgomery salivary bypass tube, Head and neck cancer, Hypopharyngeal reconstruction, Retrospective

## Abstract

**Background:**

Hypopharyngeal reconstruction after salvage pharyngolaryngectomy results in high postoperative morbidity. The use of salivary bypass tubes can reduce pharyngocutaneous fistula (PCF) formation. The influence of bacterial colonization has not been described in literature.

**Methods:**

Bacterial swipes from 26 consecutive patients reconstructed after laryngopharyngectomy in combination with Montgomery salivary bypass tubes (MSBT) were analyzed in regards to PCF formation.

**Results:**

PCF occurred in 2 untreated primary and in 9 salvage laryngopharyngectomies, respectively. Bacterial colonization showed high rates of gram-negative pathogens and drug resistance to standard Ampicillin treatment. Type of bacteria was not associated with fistula formation. Antibiotic resistance was found in 6 out 11 patients (54%) with PCF.

**Conclusions:**

We identified high rates of antibiotic-resistant Gram-negative pathogens on MSBT. Although not statistically significant, PCF were found more frequently in drug-resistant patients. Bacterial colonization of hypopharyngeal reconstructions should therefore be taken into account for perioperative prophylaxis.

## Introduction

Hypopharyngeal reconstruction after extensive oncologic resections of the larynx and hypopharynx depict a vast impact on postoperative morbidity. A variety of local and free flaps have been described to diminish fistula formation, strictures, prolonged hospitalization or delayed initiation of adjuvant therapy. However, in patients with preoperative radiotherapy the fistula rate remains at 10–50% [1–3]. The most frequently used flaps are the radial forearm free flap (RFFF), the anterolateral thigh flap (ALT) and the pectoralis major myocutaneous flap (PMMF) with a low donor-site morbidity [4–6]. Several studies show improved results by placing salivary bypass tubes beyond the pharyngoesophageal anastomosis to prevent direct contact of saliva causing postoperative fistula formation and delayed wound healing [7, 8].

On the other hand microbial colonization of Provox voice prosthesis has been shown to be associated with reduced device lifetime. The most frequently described yeast species on voice prostheses were *Candida albicans*,* Candida krusei* and *Candida tropicalis*. Bacterial colonization, however, included *Staphylococcus aureus, Pseudomonas aeruginosa, Escherichia coli, Proteus mirabilis, *and* Streptococcus agalactiae* [9].

Bacterial colonization of surgical sites in the pharynx and the larynx has been described to be highly sensitive to antibiotic treatment. An association with healing complications, however, has not been described yet [10]. We therefore examined the clinical significance of bacterial colonization of Montgomery salivary bypass tubes (MSBT) on pharyngocutaneous fistula (PCF) formation in patients after hypopharyngeal reconstruction.

## Material and methods

### Patients

26 consecutive patients treated for primary or recurrent laryngeal or hypopharyngeal squamous cell carcinoma with laryngectomy or pharyngolaryngectomy at the Department of Otorhinolaryngology, Head and Neck Surgery of the Medical University of Vienna, Austria between January 2012 and April 2016 were included in this study. Patients were reconstructed with radial forearm free flap (RFFF), serratus anterior free flap (SAFF) or pectoralis major myocutaneous flap (PMMF) or a combination of two flaps in case of circumferential defects. MSBT were inserted during surgery. Socio-demographic and clinicopathologic data, history of radiotherapy and outcome data were obtained from hospital medical reports. Bacteriology swipes from the MSBT were taken at the time of removal. Additional swipes were taken from fistulas when detected. Bacterial colonization was correlated with postoperative complications including flap failure, fistula and stricture formation, bleeding, delayed wound healing and elongated hospitalization.

The institutions’ clinical department for microbiology performed bacteriology analysis and drug-resistance patterns according to standard protocols. This study was approved by the ethics committee of the Medical University of Vienna (Ref. No. 1375/2012).

### Surgery

Laryngectomy or pharyngolaryngectomy was performed according to standard operation procedures after panendoscopy and histologic verification. Neck dissections were performed prior to tumor resection and reconstruction. A two-team approach was used in cases of free flap reconstruction. The size of the flap was measured after tumor resection. Harvesting of RFFF, PMMF and SAFF was performed as described previously [11–13]. PMMF was used with a skin island to reconstruct pharyngeal defects with a mean size of 5 × 6 cm. The mean size of the rectangular shaped RFFF was 8 × 11 cm (range 7–12 cm).

MSBT were placed intraoperatively from the base of the tongue past the distal anastomosis of the hypopharyngeal reconstruction. The surgeon selected the MSBT size (12–18 mm) by matching it to the internal diameter of the proximal esophagus. The MSBT was thereafter fixated by two nasogastric feeding tubes, which were placed through the MSBT and sutured to the nasal columella to prevent accidental displacement. In case of circumferential defects, flaps were wrapped around it followed by reconstruction using the t-shaped Connell suture as well as a two-layered closure with the pharyngeal constrictor muscle.

Arteries for free flap anastomosis included the superior thyroid artery in 4 cases and the facial artery in 6 cases. Recipient veins included the facial vein in 4 cases, the external jugular vein in 4 cases and the internal jugular vein in 2 cases. All RFFF and PMMF skin island donor sites were closed using skin grafts from the lower extremities. Tube feeding was started on the first postoperative day until barium swallow examination between the 10th and 12th postoperative days excluded pharyngocutaneous fistulas (PCF) and the MSBT could be removed.

Demographic and pathologic data were expressed as mean values with standard deviation and summarized using descriptive statistics. Recurrence and survival rates were calculated from the date of first diagnosis to the event of interest.

## Results

### Demographics

Our study cohort of 26 consecutive patients comprised 3 female and 23 male patients with a mean age of 64 years (range 50–78 years). 16 patients were treated for laryngeal carcinoma whereas 8 patients for hypopharyngeal carcinoma and 2 patients for recurrent disease of the neopharynx. T-stage included 2 patients with stage II, 7 with stage III and 16 with stage IV disease, respectively. Pathology included primary squamous cell carcinomas (SCC) in 5 patients (19%), recurrent SCC in 16 patients (54%). Recurrent SCC was found in the larynx in 14 patients and in the hypopharynx in 2 patients (Table 1). Two patients were treated for recurrent SCC in the neopharynx after prior laryngectomy, respectively. 15 patients with recurrent SCC had undergone prior radiotherapy whereas 3 patients had undergone transoral laser resection of the primary tumor. Patients with primary SCC were treated with postoperative radiotherapy. 12 patients (46%) were smokers and 9 patients (35%) were reported with regular alcohol consumption.

**Table 1:** Summary of patient demographics, history, staging, hospitalization and reconstructive procedure in 26 head and neck cancer patients after partial or total pharyngectomy

	With MSBT (*n* = 19)	Without MSBT (*n* = 7)	Total (*n* = 26)
Mean age (range)	64 (50–78)	61 (53–70)	64 (50–78)
Localisation			
Larynx	12 (63)	4 (57)	16 (61)
Hypopharynx	5 (26)	3 (43)	8 (31)
Neopharynx	2 (11)	0	2 (8)
T-stage			
T2	3 (16)	0	3 (11)
T3	4 (21)	3 (43)	7 (27)
T4	12 (63)	4 (57)	16 (62)
Prior radiotherapy	14 (74)	5 (71)	19 (73)
Mean hospital stay (days, range)	40 (11–116)	27 (7–52)	36 (7–116)
Reconstruction			
PMMF	7 (37)	4 (57)	11 (43)
RFFF + PMMF	4 (21)	0	4 (15)
SAFF	4 (21)	1 (14)	5 (19)
No flap	4 (21)	2 (29)	6 (23)

Number of patients (%) except stated otherwise

*MSBT* Montgomery salivary bypass tube, *PMMF* pectoral major myocutaneous flap, *RFFF* radial forearm free flap, *SAFF* serratus anterior free flap

### Hypopharyngeal reconstruction

**Fig. 1:**
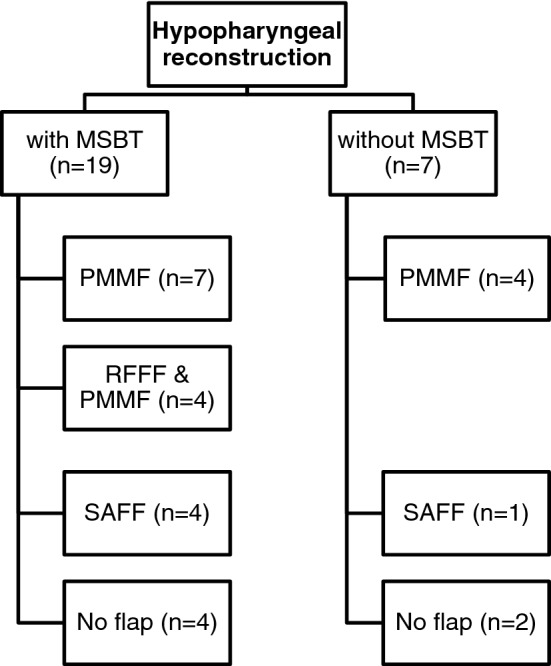
Flowchart diagram showing hypopharyngeal reconstruction with or without the Montgomery salivary bypass tube (MSBT) and regional or free flaps

Free flap selection was decided after carcinoma resection according to the size of the defect and the availability of soft tissue. The RFFF was used in combination with the PMMF for recurrent disease of the neopharynx after prior laryngectomy or patients with hypopharyngeal SCC (4 patients). The PMMF alone was used in 11 patients. In 6 patients with recurrent SCC (4 laryngeal, 2 hypopharyngeal) the SAFF were used. In 6 cases no flap was used when primary closure was feasible (Table 1). MSBT was used in 19 patients (Fig. 1). 14 of these patients had undergone prior radiotherapy. Ampicillin with sulbactam was used as standard peri- and postoperative prophylaxis. Three patients received clindamycin due to intolerance of penicillin.

## Bacterial colonization

The MSBT was placed during the operation with the proximal flange at the base of the tongue and the distal portion past the inferior anastomosis. It was fixed to nasogastric rubber tubes and sutured to the anterior nasal septum. The mean time until removal was 17 days with a range of 5–30 days because of delayed wound healing or PCF formation.

All patients received peri- and postoperative antibiotic prophylaxis (22 patients ampicillin and sulbactam, 3 patients clindamycin, 1 patient tygacil). Bacterial swipes were taken at the time of removal. Bacterial colonization of MSBT consisted of *Klebsiella pneumoniae, Klebsiella oxytoca, Pseudomonas aeruginosa, Escherichia coli *and* Viridans streptococcus*. Pathogens found in PCF included *Staphylococcus aureus, Enterobacter aerogenes, Actinomyces turicensis *and* Serratia marcescens*. Drug resistance patterns showed that in 9 patients Gram-negative bacteria were not sensitive to ampicillin. One patient treated with clindamycin showed resistant *Viridans streptococci*. No correlation between type of bacterial colonization and fistula formation or delayed wound healing was found. However, in 6 out of 11 patients (54%) with PCF antibiotic-resistant bacteria were found compared to 2 drug-sensitive patients. In 3 patients, drug-resistance patterns were not available (Table 2).

**Table 2:** Formation of pharyngocutaneous fistulas (PCF) according to type of bacterial colonization of 19 Montgomery salivary bypass tubes (MSBT) and 6 patients without MSBT after hypopharyngeal reconstruction

Bacteriology	PCF	No PCF
*Klebsiella pneumoniae*	3	0
*Klebsiella oxytoca*	0	2
*Pseudomonas aeruginosa*	1	1
*Escherichia coli*	0	1
*Staphylococcus aureus*	1	0
*Enterobacter aerogenes*	1	1
*Actinomyces turicensis*	1	0
*Serratia marcescens*	1	0
*Viridans streptococcus*	0	1
Antiobiotic resistance	6	2
N/A	3	9
Total	11	15

*PCF* pharyngocutaneous fistula, *N/A* not available

### Outcome

There were no perioperative deaths. The mean duration of hospitalization in the entire study population was 29 days (range 7–116 days). Complications occurred in 58% of patients (*n* = 15). These included flap failure (*n* = 2), PCF (*n* = 11), stricture (*n* = 2), bleeding or hematoma (*n* = 7) and delayed wound healing (*n* = 6) (Table 3). Two SAFF had to be replaced with PMMF due to flap failure. In one patient flap necrosis occurred 10 days after surgery and was caused by major postoperative delirium. In the second patient, arterial thrombosis distal to the anastomosis was observed intraoperatively and could not been redeemed by microvascular revision. Hence the flap was replaced during the same operation by a PMMF. Patients with postoperative complications stayed 45 days (range 7–116 days) compared to 24 days (range 11–44 days) in patients without complications. Patients with a MSBT stayed 40 days (range 7–52 days) compared to 27 days (range 11–116 days) in patients without MSBT.

**Table 3:** Incidence of postoperative complications (*n* = 29) according to clinical parameters

	Flap failure	Fistula	Stricture	Bleeding hematoma	Wound healing
Primary SCC (*n* = 5)	1 (20)	2 (40)	0	0	3 (60)
Recurrent SCC (*n* = 21)	2 (10)	9 (43)	2 (9)	7 (33)	3 (14)
Prior RT (*n* = 19)	2 (11)	9 (47)	2 (11)	5 (26)	3 (16)
Flap type					
PMMF (*n* = 11)	1 (9)	4 (36)	0	2 (18)	3 (27)
RFFF + PMMF (*n* = 4)	0	1 (25)	1 (25)	2 (50)	1 (25)
SAFF (*n* = 6)	1 (17)	2 (33)	1 (17)	2 (33)	1 (17)
No flap (*n* = 5)	0	4 (66)	0	1 (17)	1 (17)
With MSBT (*n* = 19)	2 (11)	8 (42)	2 (11)	6 (32)	4 (21)
Without MSBT (*n* = 7)	0	3 (43)	0	1 (14)	2 (29)
Total (*n* = 29)	2 (9)	11 (42)	2 (7)	7 (27)	6 (23)

Number of complications (%) except stated otherwise

*SCC* squamous cell carcinoma, *RT* radiotherapy, *PMMF* pectoral major myocutaneous flap, *RFFF* radial forearm free flap, *SAFF* serratus anterior free flap, *MSBT* Montgomery salivary bypass tube

In 11 patients (42%) postoperative PCF were found at an average of 23 days (range 7–40 days) after surgery. In particular, PCF occurred in 2 out of 5 (40%) with primary and 9 out of 19 (47%) salvage laryngopharyngectomies. Eight of these patients had elevated CRP levels at the time of fistula detection or surgical revision. Four patients had been reconstructed with a PMMF, one patient with RFFF and PMMF and 2 patients with SAFF. In 4 patients with fistula formation no flap had been used (Table 3). Fistula revision was performed by refreshment of the tissue, suture of the leakage and application of fibrin sealant. In 5 patients an additional PMMF was used for fistula closure. One of these patients developed an oesophagotreacheal fistula and was treated with a permanent G-tube.

Seven patients had to be revised due to postoperative hematomas of the flap donor site or bleeding of the primary site. Proximal tube migration was reported in one patient but was easily repositioned in local anesthesia. Eleven patients (42%) were able to resume oral alimentation, 9 patients (35%) needed a G-tube. The mean follow-up of all patients included in this study was 15 months. At the end of the follow-up period, 15 patients were alive without evidence of disease, 6 patients were alive with evidence of disease and 5 patients died (3 died due to disease, 2 died of other cause). One of these patients died 3 weeks after operation due to heart failure in the hospitalization room.

## Discussion

Postoperative complications such as delayed wound healing and fistula formation after laryngectomy and pharyngectomy are common and depict a great socioeconomic challenge. The insertion of MSBT in local or general anesthesia has been described a useful tool to treat pharyngocutaneous fistulas after laryngectomy [7, 14]. Even though the prophylactic use of MSBT with fasciocutaneous flaps has been recommended to prevent severe complications, the rate of fistulas remains high in pre-treated head and neck cancer patients [15]. A study on 103 patients with hypopharyngeal defects showed that intraoperative placement of salivary bypass tubes reduces the fistula formation from 22 to 7%. However, in patients with secondary reconstruction the fistula rate was still given at 35% [16]. It seems that placing salivary bypass tubes beyond the pharyngoesophageal anastomosis and thereby restricting the amount of direct contact of saliva with the mucosa does not prevent fistula formation in all patients. Poor oral hygiene, cigarette smoking and poor nutritional status account for microbial colonization [17]. There might be other factors leading to mechanical decay of the tube itself or the pressure of the tubes weakening the mucosa of the neopharynx. We therefore aimed to identify the type of bacterial colonization on MSBT in patients undergoing laryngectomy with partial or total pharyngectomy for laryngeal or hypopharyngeal SCC and to analyze the influence on the rate of postoperative complications.

In 15 out of 26 consecutive patients 29 postoperative complications occurred, which represents an overall rate of 58%. This is line with a previous publication by Lopez and co-workers who reported a complication rate of 53% in 55 patients reconstructed with RFFF or ALT and MSBT [8]. In particular we found 22 complications in 10 patients with MSBT compared to 6 complications in 5 patients without MSBT. Surprisingly, the use of MSBT did not prevent fistula formation in our study population. Flap failure, PCF, stricture, bleeding or hematoma and delayed wound healing were found in both groups. The development of complications, however, led to prolonged hospitalization. The mean duration of hospitalization in patients with complications was 45 days compared to 24 days without complications. Interestingly, also patients with MSBT stayed 40 days compared to only 27 days without MSBT.

A study by Fusconi and colleagues investigated the in vivo biofilm formation on Montgomery tracheal Safe-T-Tubes in regards to patient discomfort and clinical failure [18]. They concluded that mechanical decay might partly be explained by the release of inflammatory enzymes.

Bacterial colonization of the MSBT in our study included *Klebsiella pneumoniae, Klebsiella oxytoca, Pseudomonas aeruginosa, Escherichia coli, Staphylococcus aureus, Enterobacter aerogenes* and resident oropharyngeal flora. This is in accordance to a study by Yang and co-workers who correlated contamination of surgical wounds with postoperative infections. They found *Pseudomonas aeruginosa* and *Enterococcus faecalis* to play a greater role than typical oral commensals. They describe a rapid increase in resistance to clindamycin [19]. A change of oral colonization towards Gram-negative species during hospitalization is known [20]. In our study, type of bacteria did not correlate with postoperative complications such as PCF and stricture formation. Interestingly, however, we observed a high proportion of Gram-negative bacteria including *Pseudomonas aeruginosa* on MSBT and postoperative fistulas. In particular, we found high rates of ampicillin-resistant Gram-negative pathogens.

According to elevated CRP levels in our patients, at the time when the swipes were taken, we assume an infectious cause for PCF formation. We therefore suggest early bacterial swipes a standard routine in patients after hypopharyngeal reconstruction. Hence the standard antibiotic prophylaxis could be changed from ampicillin to piperacillin.

We acknowledge the heterogeneity of our study population. In these difficult-to-treat patients we observed an overall postoperative complication rate of 58%. There was no correlation between bacterial colonization of MSBT. However, we observed high rates of Gram-negative pathogens and drug resistance to the standard antibiotic therapy at our institution. Biofilm research has been shown to be more complex than identifying individual species due to microbial interaction [21].

Montgomery salivary bypass tubes in combination with free flaps have been described as successful in the reconstruction of the hypopharynx. This is the first study to examine the clinical relevance of bacterial colonization of MSBT in head and neck cancer patients. We observed high rates of Gram-negative bacteria with drug resistance to standard antibiotic prophylaxis. Although in a limited number of patients we observed more PCF in drug-resistant patients. Increasing rates of resistance should be taken into account for future antibiotic prophylaxis and indicate the need for an effective alternative. We therefore need more clinical studies to improve the perioperative antibiotic treatment in high-risk patients.
